# Increasing varicella incidence rates among children in the Republic of Korea: an age–period–cohort analysis

**DOI:** 10.1017/S0950268819001389

**Published:** 2019-07-22

**Authors:** Young Hwa Lee, Young June Choe, Sung-Il Cho, Ji Hwan Bang, Myoung-don Oh, Jong-Koo Lee

**Affiliations:** 1 Department of Epidemiology, Seoul National University School of Public Health, Seoul, Republic of Korea; 2 Division of Pediatric Infectious Diseases, The Warren Alpert Medical School of Brown University, Providence, RI, USA; 3 Division of Infectious Diseases, Seoul Metropolitan Government-Seoul National University Boramae Medical Center, Seoul, Republic of Korea; 4 Department of Internal Medicine, Seoul National University College of Medicine, Seoul, Republic of Korea; 5 Department of Family Medicine, Seoul National University College of Medicine, Seoul, Republic of Korea

**Keywords:** Age–period–cohort, chickenpox, incidence, varicella

## Abstract

In the Republic of Korea, despite the introduction of one-dose universal varicella vaccination in 2005 and achieving a high coverage rate of 98.9% in 2012, the incidence rate has been increased sevenfold. This study aimed to investigate time trends of varicella incidence rate, assessing the age, period and birth cohort effects. We used national data on the annual number of reported cases from 2006 to 2017. A log-linear Poisson regression model was used to estimate age–period–cohort effects on varicella incidence rate. From 2006 to 2017, the incidence of varicella increased from 22.5 cases to more than 154.8 cases per 100 000. Peak incidence has shifted from 4 to 6 years old. The estimated period and cohort effects showed significant upward patterns, with a linear increasing trend by net drift. There has been an increase in the incidence among the Korean population regarding period and cohort despite the universal vaccination of varicella vaccine. Our data suggest the need for additional studies to address the current gap in herd immunity.

## Introduction

Varicella is an acute infectious disease caused by the varicella-zoster virus. It is highly communicable, with secondary attack rates >90% among susceptible individuals [1, 2]. The varicella vaccine, which became available in the early 1980s, conferred excellent immunogenicity against varicella infection. Countries such as the USA, Germany and Taiwan which adopted varicella vaccination programme experienced a reduction in the incidence rate of varicella [3–5].

In the Republic of Korea, one dose of varicella vaccination was introduced to the National Immunization Program (NIP) in 2005. The mandatory varicella vaccination was recommended for 12–15 months old infants. Imported and domestic live attenuated vaccines are available in Korea. The former is based on Oka strain and used widely in many countries and the latter is based on MAV strain which is isolated from a Korean boy and is predominantly used in Korea. However, the incidence rate of varicella has yet to decline and, in fact, has been continuously rising, from 22.5 per 100 000 persons in 2006, to 154.8 in 2017 [6], despite the vaccine coverage reaching up to 98.9% in 2012 [7].

The age, period and cohort (APC) effects may provide an important epidemiologic clue to elucidate the current gap in immunity. Age effects are associated with different age groups, period effects affect all ages simultaneously over time, while cohort effects are related to changes among groups of individuals born in the same year. For instance, age effects imply the biological susceptibility of people of a specific age, period effects reflect environmental changes, diagnostic efficiency or changes in surveillance practice and cohort effects represent early exposure to risk factors. The APC analysis has been used to study the time trends in the incidence of infectious diseases [8–11]. The model separates the time trends into the effects of age, period and cohort.

In this study, we used the APC model to obtain a better understanding of these effects on the incidence of varicella in Korea. The results might provide guidance for future epidemiological research and may implicate for better surveillance and vaccination policies.

## Materials and methods

### Data collection

In Korea, the national surveillance system established in 2001 consists of case-based national infectious disease data. Varicella has become nationally notifiable since July 2005 and reporting is mandatory in which medical doctor, an oriental medical doctor and the head of the public health centre or commander of a unit belonging to the Army, Navy or Air Force are obliged to report the incidence of the disease. The obliged have to immediately report a confirmed or probable case to the head of competent public health centre and then the case was finally reported to the Korea Center for Infectious Disease Control and Prevention (KCDC) through the web-based reporting system. In this study, to use full-year data on the annual varicella incidence, we obtained the National Notifiable Disease Surveillance System data from January 2006 to December 2017. Population statistics were available from the Korean National Statistics Office. The person-years of observation were tabulated into 1-year classes for ages 0–12 and for the calendar period 2006–2017.

### Statistical analysis

The APC model was used to estimate the age, period and cohort effects. The standard APC model assumes that the observed number of varicella infections follows a Poisson distribution and that the incidence rates are a multiplicative function of age, cohort and period, such that the logarithm of the rates is an additive function of the parameters [12–15]. The log age-specific rate *λ* (*a*,*p*) at age *a* in period *p* for people in cohort *c* = *p*−*a* is as follows:
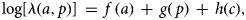
where *a*, *p* and *c* denote the mean age, period and cohort, respectively, for the observational units and *f*, *g* and *h* are parametric functions. The exact linear dependence of the regression variables (*c* = *p*−*a*) causes identifiability problem [15]. To decompose these three components into linear and non-linear parts and to obtain estimable functions such as the log-linear trend by period and cohort, we adopted the APC models proposed by Rosenberg [16], and conducted APC analysis by the APC Web Tool [17].

This online web tool provides ‘net drift’, indicates the annual percentage change of the expected age-adjusted rates over time (period and cohort); ‘local drift’, the annual percentage change of the expected age-specific rates over time; ‘longitudinal age curve’, the expected age-specific rates in reference cohort adjusted for period effects; ‘period (or cohort) rate ratio (RR)’, the age-adjusted relative risk in each period (or cohort) *vs.* to reference one. The corresponding Wald test was used to determine significance.

### Ethics statement

The present study protocol was reviewed and approved by the Research Ethics Board of the Seoul National University (IRB No. E-1609-030-789).

## Results

### Descriptive data

**Fig. 1.**
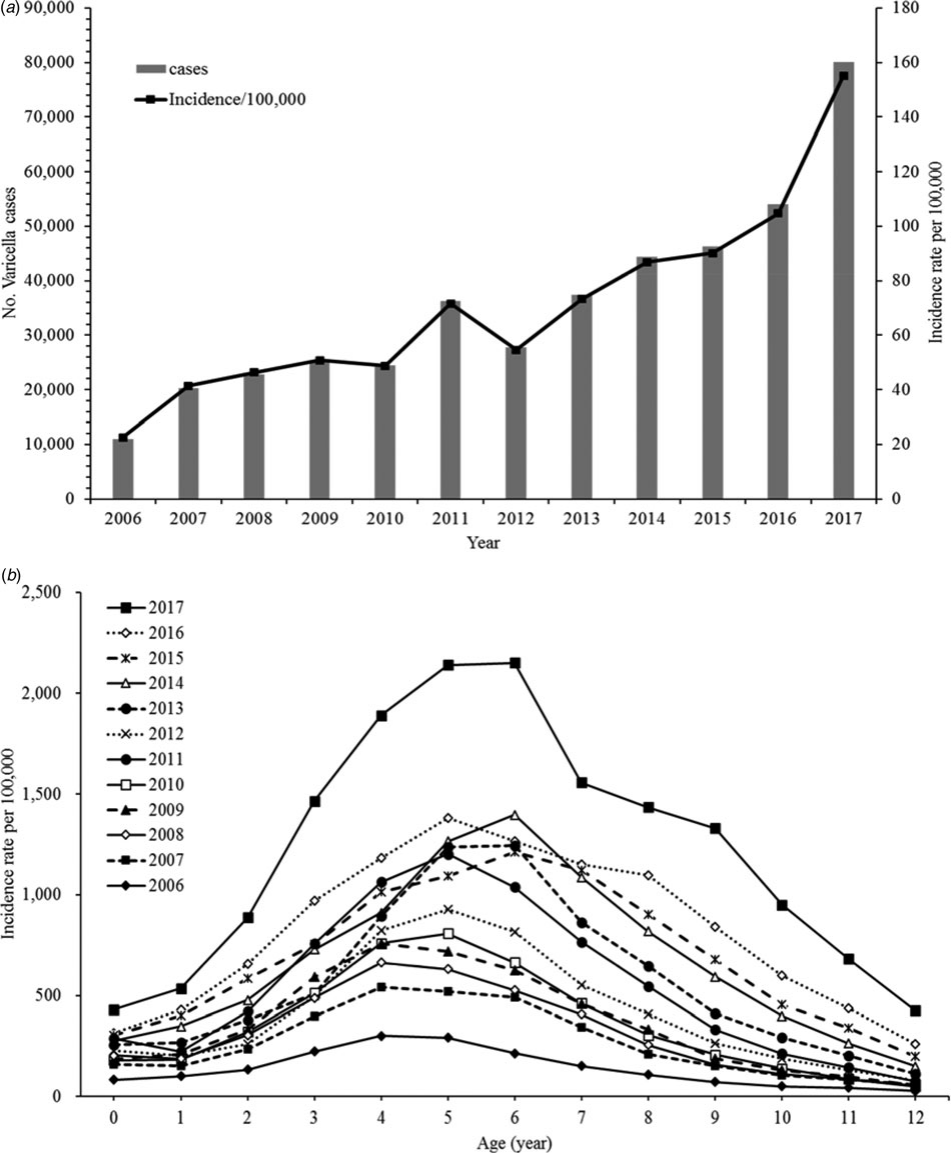
(a) Age-standardised incidence rates of varicella, 2006–2017. (b) Age-specific incidence rates of varicella, 2006–2017.

Varicella incidence and age-standardised incidence rates have shown a diagonally upward trend between 2006 and 2017 (Fig. 1a). The incidence rate stratified by period also increased for almost all age groups (0–12 years old). The rates peaked between ages 4 and 6 years and we observed a drop-off in older ages (Fig. 1b).

**Fig. 2.**
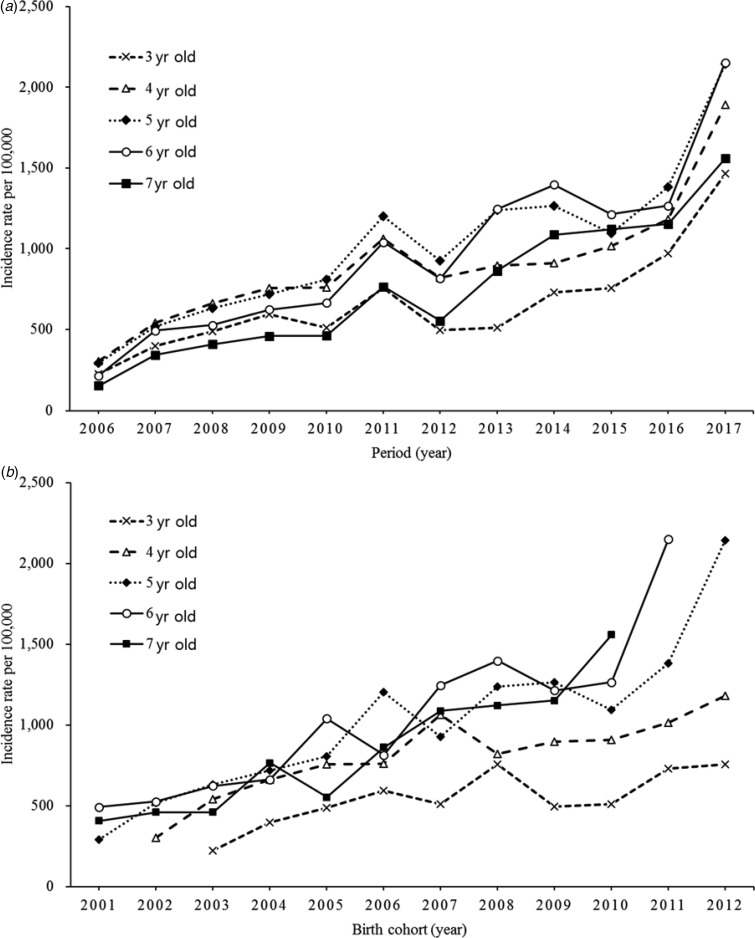
(a) Age-specific incidence rates of varicella by period, 2006–2017. (b) Age-specific incidence rates of varicella by birth cohort, 2006–2017.

An increasing tendency of higher varicella incidence rates with later periods was determined for each age group (Fig. 2a). The peak of incidence rate shifted from 4 years of age during 2006–2009, to 5 during 2010–2012, and to 6 during 2013–2017 (except in 2016), reflecting an age shift. The cohort curves also showed an increasing trend with later birth cohorts, especially in ages 5 and 6 years (Fig. 2b). The age-specific rates were proportional to both period and birth cohorts.

### Age–period–cohort analysis

**Fig. 3.**
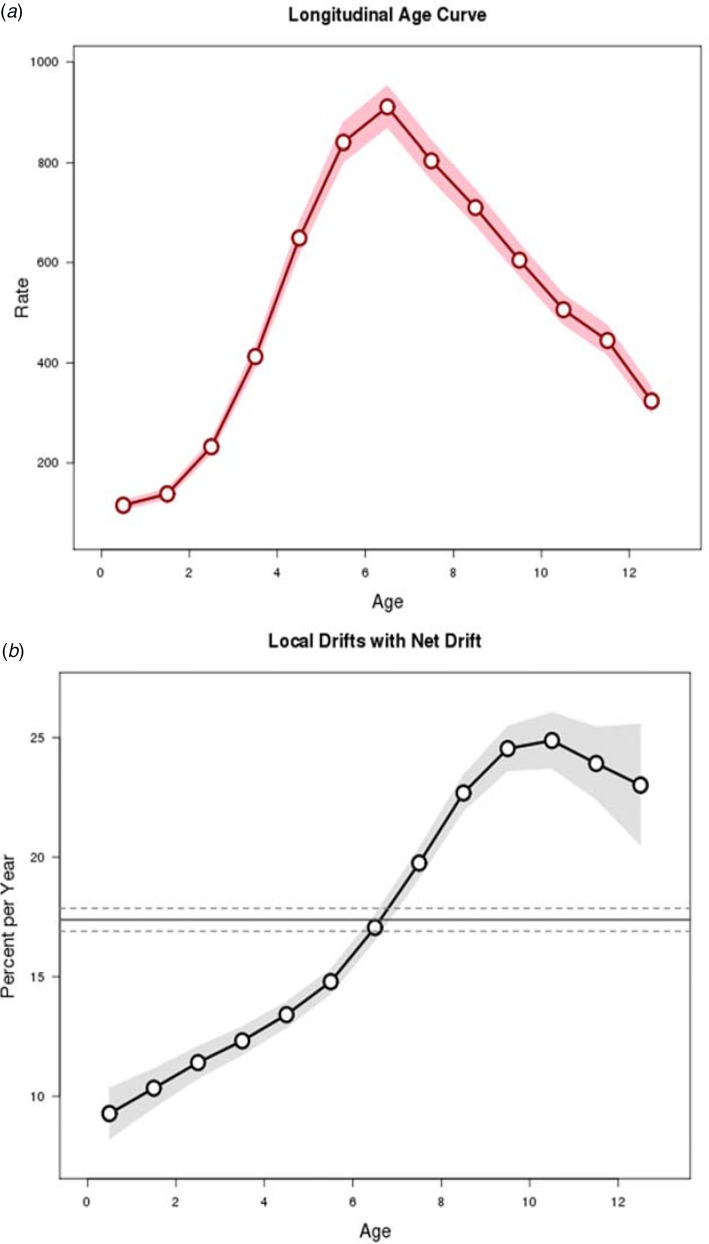
Longitudinal age curve and drifts (net drift and local drifts) obtained by age–period–cohort analyses for the incidence rate of varicella and the corresponding 95% confidence intervals, 2006–2017.

**Fig. 4.**
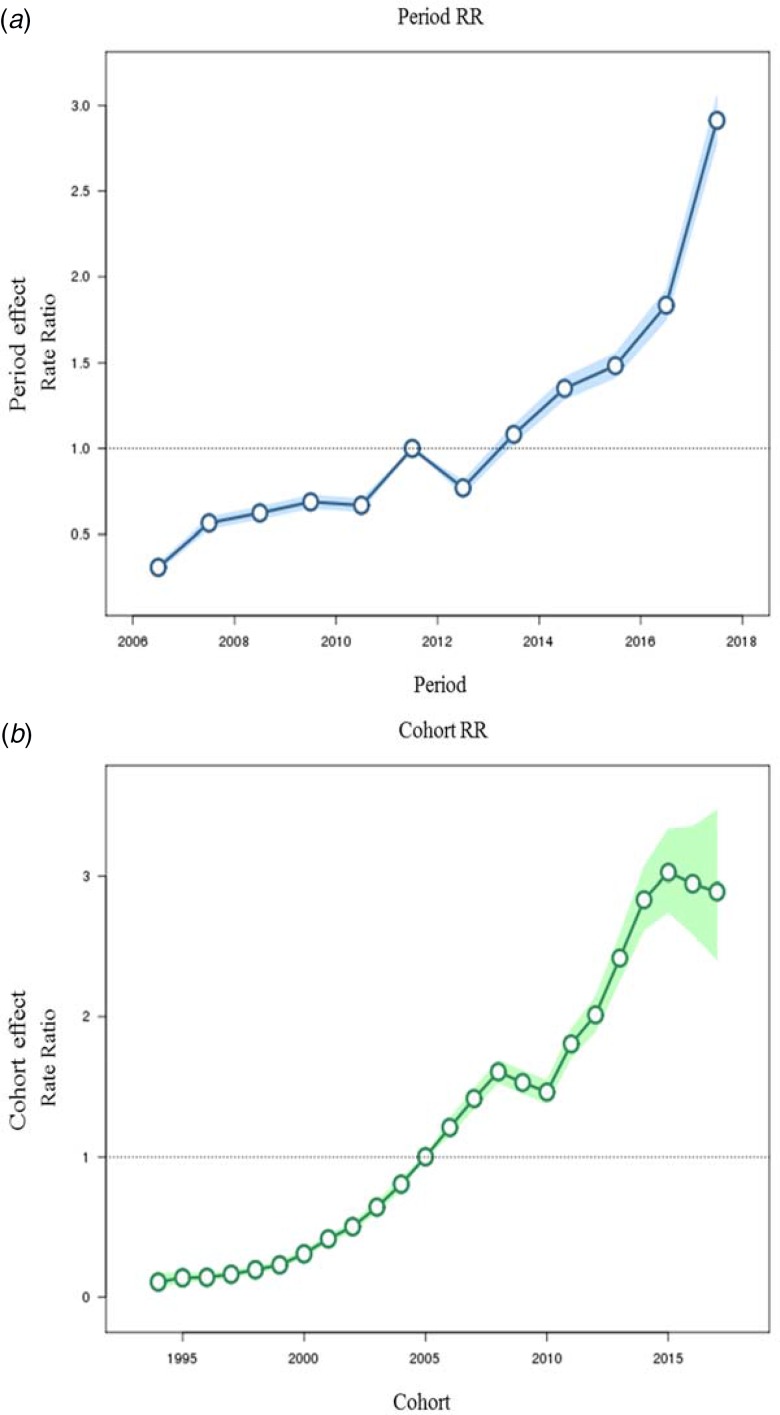
Period and cohort effects obtained by age–period–cohort analyses for the incidence rate of varicella and the corresponding 95% confidence intervals, 2006–2017.

The age, period and cohort effects are presented in Figures 3 and 4. The longitudinal age curve of varicella incidence rate displays the risk increased to peak at the ages 6–7 years and then declined thereafter (Fig. 3). The net drift, which indicates the annual percentage change of the estimated age-adjusted rates over time, was 17.4 and the curves of local drift, which reflect the annual percentage change of the estimated age-specific rates over time, showed an upward trend with a peak at the age of 10–11 years.

The estimated period and cohort RRs showed similar increasing patterns; however, period RR was dramatically elevated in 2017 while cohort RR slightly decreased after the year 2015 (Fig. 4).

Wald tests suggested both period and cohort effects were statistically significant (*P* < 0.05 for all).

## Discussion

Despite the implementation of the universal varicella vaccination programme in July 2005, there was an increase in the incidence rate of varicella between 2006 and 2017 in Korea. Our finding demonstrated that the period and cohort effects showed an upward trend and the age peak in the incidence rate shifting from 4 to 6 years old. This may indicate that a universal one-dose varicella vaccination in Korea has not been successful in preventing varicella zoster virus.

These findings contradict the observations in other countries. In the USA, routine one-dose vaccination of all children between the ages of 12 and 18 months was implemented in 1996 and has resulted in decreases in the incidence from 1.1–3.8 cases per 1000 population between 1990 and 1994 to 0.3–1.0 cases between 1999 and 2001 [3]. The vaccine effectiveness of one-dose vaccination was estimated to be 85% (95% CI 78–90%; *P* < 0.001) [18]. Elsewhere, introduction of one-dose vaccine to the NIPs has led to decreases in the incidence even when vaccination coverage is suboptimal. In Germany, where a routine varicella vaccination programme was introduced in 2004, vaccination coverage between 2006 and 2011 was only 38–68%, whereas the number of cases decreased by 67%: from 6.6 per 1000 patients in 2006–2007 to 2.2 in 2010–2011 [4]. In Taiwan, implementation of a national free vaccination programme led to an increase of vaccination coverage from <10% before 2003 to 80% in 2004; in addition, there was a decrease in the age-standardised incidence rates from 7.2 in 2004 to 3.2 cases per 1000 person-years in 2008 [5].

Our data may be explained by primary and/or secondary vaccine failure. Primary failure relates to the failed mounting of the immune system to produce antibodies initially [19]. A prospective case-based study conducted from 2006 to 2007 in Korea showed almost no impact of varicella vaccine introduction, possibly due to insufficient immunogenicity of vaccine based on MAV strain [20]. The vaccine immunogenicity estimated in the case–control study was 54% (95% CI 0.1–2.1), and the classical fluorescent antibody to membrane antigen (FAMA) assay revealed that the seroconversion rate was 76.7%.

Secondary failure refers to the waning of vaccine-induced immunity over time [19]. Recent studies suggest that one-dose varicella vaccination has limited effectiveness to prevent outbreaks in mass gatherings or schools. In the USA, after the introduction of varicella vaccine, there was a substantial difference in the vaccine's effectiveness in the first year after vaccination (97%) and in years 2–8 after vaccination (84%, *P* = 0.003) [21]. Another retrospective cohort study involving students attending elementary school suggested that 99% of one-dose vaccination coverage was not sufficient to prevent varicella outbreaks [22]. A longitudinal seroprevalence study in Korea showed a progressive decrease of the seropositivity rates following vaccination: 65% at age 1 year, 59% at age 2 years, 53% at age 3 years and 49% at age 4 years [23]. One may also postulate that one dose of varicella vaccination may result in secondary vaccine failure, or waning of immunity over time. In one study from Korea that determined seroprevalence using the FAMA and ELISA assays showed a decreasing trend in geometric mean titres according to the interval since vaccination over time [24]. A cross-sectional study in four Korean hospitals tested the seroprevalence of 887 patients in which overall 87.6% had anti-VZV IgG antibody [25]. The prevalence of anti-VZV IgG antibody was 75% during the first 3 months of age, but decreased to 13.6% at 12 months of age. Anti-VZV IgG antibody prevalence increased first at 1–2 years of age and then at 5–6 years of age. It is unclear if the increase in 5–6 years of age resulted from second vaccinations which are not supported by the government, or was from boostered from the natural transmission of the virus. But overall, the seroprevalence rate exceeded 90% in subjects over 11 years of age. Another study investigating residual serum from diagnostic laboratories in Korea showed an increase in seroprevalence by age as 67.3% of subjects 1–4 years of age were seropositive whereas 94.2% of subjects 10–14 years of age were seropositive [26]. The decreasing trend of antibody level may explain the continuing increase of varicella in all given cohorts despite the introduction of the vaccine into the national immunisation programme. According to Lee *et al*. [27], varicella vaccine effectiveness in Korea sharply declined after the third year of vaccination. Considering Korea's high rate of vaccine coverage, most of the varicella incidence is associated with a breakthrough case. The age peak shifting detected in Korea, in spite of a rise in the incidence rate of varicella, could be associated with secondary failure. The age shift usually occurs with a decrease in the incidence rate because one-dose varicella vaccine applied to younger children about 12–18 months of age reduces the exposure to circulating varicella zoster virus. The varicella vaccine, however, is merely effective in the early years, but, later, the incidence of breakthrough infection jumps as immunity rapidly wanes over time. Furthermore, if the vaccine has a positive effect in the attenuation of disease severity despite that the vaccination failed to protect against varicella incidence, this could lead to a growing number of breakthrough cases as being unsuccessful in isolating patients with mild symptoms. A recent cross-sectional population-based study [28] on 1008 reported varicella cases among children during 2015–2017 demonstrated that the risk of severe illness (mild against moderate-to-severe) was significantly decreased by 0.570 in the breakthrough group compared with the unvaccinated group. The recent increase in varicella notification may partly be associated with these attenuated varicella cases.

Historical context is important in interpreting the data. In Korea, a varicella vaccine was first licensed and distributed in the private market since 1988 [29]. There is no accurate data on vaccine coverage rate in the 1990s, but given the annual production volume over 500 000 doses, which is larger than the annual birth cohort of 400 000–500 000, the one-dose coverage rate may have been sustained for more than decades. The first survey to measure the vaccination coverage for varicella at regional rates was conducted in 2000 and was based on 850 children for whom vaccination record books were available. The survey revealed an overall varicella vaccination coverage of 72.5% [30]. In a subsequent coverage study, carried out in 2012 by face-to-face interview-based questionnaire survey among randomly selected 3393 children aged 19–83 months, coverage with the one-dose varicella vaccine was 98.9% [7]. Given the high vaccination coverage in Koreans prior to the introduction to the NIP, the programme may have not impacted the incidence of varicella greatly.

An increase in notification rate due to reporting bias partly contributes to the rapid increase of varicella notification. In particular, observed notifications in early surveillance periods might be under-reported because national surveillance of varicella was started in 2005 when varicella vaccine was introduced to the NIP and the reporting system may not have been fully active during its initial stages. Since then, the notification system has improved to be internet-based in 2009 for clinics and hospitals in order to enhance the reporting rate by frontline doctors [31]. Also, amendment in the Infectious Disease Control and Prevention Act in 2016, which expanded reporting parties from medical institutions to include diagnostic laboratory agencies, might enhance the reporting rate as in the case of scarlet fever [32]. However, increased notification would have been applied similarly to all cases and reflected as the rise of period effect over the years. Even after taking this period effect into consideration, more recent birth cohorts still showed a higher risk (Fig. 4), which cannot be fully explained by strengthened surveillance.

Our study had a limitation that lack of the incidence data before the implementation of one-dose varicella vaccine programme made it hard to evaluate the exact effectiveness of the vaccination programme. In addition, relevant data such as a change in the type of vaccines and in disease severity over time were unavailable so that the association of an increase in varicella notifications with vaccine failure was not clearly explained. The current study also limited its focus on assessing the presence of cohort effects, and further analysis of possible reasons for any actual increase requires additional data and methods such as follow-up of vaccinated children, analysis of National Health Insurance claims data and mathematical modelling. Despite these limitations, the present study is unique to evaluate the APC effects in a national varicella vaccination programme. Our data indicate that individuals in all the cohorts, as well as more recently born cohorts, have a higher incidence of varicella infection, signalling a potential need for investigation on the gap of immunity.

In conclusion, there has been an increase in the incidence of varicella among the Korean children with age peak shifting from 4 to 6 years old. Our data suggest the need for further studies on primary and secondary vaccine failure against varicella in the Republic of Korea. Future studies of vaccination effectiveness may be possible with retrospective cohort design or case–control design by combining vaccination database and medical insurance claims data. Such a study will require administrative collaborations among relevant organisations. Furthermore, studies with new data collection for case–control or cohort studies may be also conducted by developing appropriate collaboration networks of clinical institutions. In addition, the adoption of a two-dose vaccination policy should be considered after the evidence of immunogenicity provided by one-dose varicella vaccination becomes more clear and effective.
